# Effects of Intravenous Versus Oral Iron Replacement on Inflammatory Markers and Serum Uric Acid in Premenopausal Women with Iron Deficiency: A Prospective Study

**DOI:** 10.3390/nu18213518

**Published:** 2026-10-26

**Authors:** Vehbi Demircan, Kübra Yazar, Hakan Eminoğlu, Yavuz Katırcılar, Abdullah Karakuş, Turgay Ulaş, Mehmet Sinan Dal, Mehmet Orhan Ayyıldız

**Affiliations:** 1 Department of Hematology, Dicle University, 21280 Diyarbakır, Turkeyorhanay21@hotmail.com (M.O.A.); 2 Department of Hematology, Dr. Abdurrahman Yurtaslan Ankara Training and Research Hospital, 06200 Ankara, Turkeydr.sinandal@gmail.com (M.S.D.); 3 Department of Hematology and Bone Marrow Transplantation Unit, Kayseri Medical Faculty, University of Health Sciences, 38030 Kayseri, Turkey

**Keywords:** iron deficiency, intravenous iron, oral iron, C-reactive protein, erythrocyte sedimentation rate, uric acid, premenopausal women

## Abstract

Background/Objectives: Iron deficiency may be associated with alterations in systemic inflammatory activity and metabolic parameters. However, whether oral and intravenous (IV) iron replacement have different effects on inflammatory markers and serum uric acid remains unclear. We compared inflammatory and metabolic responses to oral and IV iron in premenopausal women with iron deficiency. Methods: This prospective observational study included 72 oral iron recipients, 77 IV iron recipients, and 76 healthy controls. Patients were reassessed at Day 30. The primary outcome was change in C-reactive protein (CRP); changes in erythrocyte sedimentation rate (ESR) and uric acid were secondary outcomes. Additional regressions used robust standard errors, adjusting for age, baseline hemoglobin, and baseline outcome values; the uric acid model additionally included baseline creatinine. Results: Baseline CRP and ESR were significantly higher in both iron-deficient groups than in controls (all Bonferroni-adjusted *p* < 0.001). The IV iron group had more severe baseline anemia than the oral iron group (median hemoglobin: 8.60 vs. 10.70 g/dL; Bonferroni-adjusted *p* < 0.001). Increases in hemoglobin, ferritin, and transferrin saturation over 30 days were greater with IV iron (all *p* < 0.001). Median CRP changes were −0.22 mg/L with IV iron and 0.07 mg/L with oral iron (unadjusted *p* = 0.041). However, multivariable regression with robust standard errors showed no statistically significant difference in Day 30 CRP after adjustment for age, baseline hemoglobin, and baseline CRP (IV minus oral: −1.585 mg/L; 95% CI, −3.261 to 0.091; *p* = 0.064; *n* = 149). The corresponding adjusted analysis showed lower Day 30 ESR with IV iron (−2.118 mm/h; 95% CI, −3.817 to −0.418; *p* = 0.015; *n* = 91). The adjusted serum uric acid difference, additionally accounting for baseline creatinine, was not statistically significant (0.389 mg/dL; 95% CI, −0.009 to 0.787; *p* = 0.055; *n* = 149). Conclusions: IV iron was associated with greater hematologic and iron-store repletion over 30 days. Adjusted comparisons showed no statistically significant differences in CRP or serum uric acid, whereas ESR was modestly lower with IV iron, with uncertain clinical significance. Nonrandomized treatment allocation and premedication restricted to the IV group preclude attributing these findings solely to iron administration.

## 1. Introduction

Iron deficiency anemia (IDA) is the most prevalent nutritional deficiency worldwide and a major cause of anemia, particularly among women of reproductive age. Beyond its well-established hematologic consequences, iron deficiency may adversely affect cellular metabolism, mitochondrial function, oxidative balance, and immune regulation [1,2]. Iron homeostasis depends on coordinated intestinal absorption, macrophage-mediated recycling, transferrin-bound transport, and ferritin storage. The hepatic hormone hepcidin regulates systemic iron availability by binding to ferroportin and limiting iron export from enterocytes and macrophages [3]. Dysregulation of this axis contributes to distinct disease states: insufficient hepcidin activity promotes iron overload in hereditary hemochromatosis, whereas inflammation-induced hepcidin elevation restricts iron availability for erythropoiesis and contributes to anemia of inflammation [4]. Disorders of iron metabolism are also relevant to chronic kidney disease and inflammatory bowel disease, in which absolute iron deficiency may coexist with inflammation-driven functional iron deficiency. In these conditions, increased hepcidin activity reduces intestinal iron absorption and promotes iron sequestration within macrophages, limiting iron availability for erythropoiesis despite retained tissue iron stores [5].

C-reactive protein (CRP) and erythrocyte sedimentation rate (ESR) are widely used laboratory indicators of systemic inflammatory activity. CRP is an acute-phase protein that responds relatively rapidly to changes in inflammatory signaling, whereas ESR is an indirect marker influenced by plasma proteins and hematologic variables, including anemia and red blood cell characteristics [6,7]. The interpretation of inflammatory markers in iron deficiency requires caution, because ESR is influenced by anemia and erythrocyte characteristics in addition to inflammatory activity. A reduction in CRP following iron replacement has been reported in women with iron-deficiency anemia [6,8]. These observations provide a rationale for evaluating CRP and ESR during iron replacement, while considering the potential contribution of hematologic recovery to changes in ESR.

Serum uric acid, the final product of purine metabolism in humans, has also been linked to oxidative stress and inflammation. Its biological activity is complex and concentration-dependent. Although extracellular uric acid has antioxidant properties, intracellular uric acid and monosodium urate crystals may activate oxidative and inflammatory pathways, including nuclear factor-κB and the NLRP3 inflammasome [9,10]. Serum uric acid has also been associated with conventional inflammatory biomarkers, including CRP, fibrinogen, ferritin, and complement C3 [9]. Therefore, uric acid may be considered an inflammation-associated metabolic biomarker rather than a conventional acute-phase reactant. Beyond its association with inflammation, serum uric acid may be linked to iron status itself. Population-based analyses have demonstrated positive associations between ferritin and serum urate after adjustment for CRP, while Mendelian randomization findings suggest a potential effect of iron status on circulating urate [11]. An increase in serum uric acid following oral iron therapy has also been reported in young women with iron-deficiency anemia [12]. These findings provide a rationale for assessing uric acid alongside CRP and ESR during iron replacement. Their combined evaluation may help characterize whether changes in inflammatory markers are accompanied by changes in urate metabolism, without assuming that these parameters reflect identical biological processes or change in the same direction. These observations provide a rationale for examining serum uric acid during iron replacement, but they do not establish that iron administration directly alters purine metabolism or that oral and intravenous formulations have different effects on circulating urate. The different patterns of iron delivery and repletion provide a basis for comparing the two treatment regimens, while the direction and magnitude of any uric acid response remain uncertain. Serum uric acid was therefore evaluated as an exploratory secondary metabolic outcome, rather than as a direct measure of systemic inflammation or a surrogate for a presumed anti-inflammatory effect.

Persistent systemic inflammation is associated with adverse long-term health outcomes, including cardiovascular disease, diabetes mellitus, chronic kidney disease, malignancy, non-alcoholic fatty liver disease, and neurodegenerative disorders [13]. Accordingly, the early recognition and correction of potentially reversible factors contributing to inflammatory activity may be clinically relevant. Iron replacement can be administered orally or intravenously; however, these routes differ in bioavailability, speed of iron repletion, gastrointestinal exposure, and potential effects on oxidative and inflammatory pathways [1,2].

This prospective observational study aimed to compare changes in CRP, ESR, and serum uric acid from baseline to Day 30 between premenopausal women with iron deficiency receiving oral or intravenous iron replacement. Baseline comparisons with healthy controls were also performed to characterize differences in these parameters according to iron status.

## 2. Materials and Methods

### 2.1. Study Design and Participants

This prospective observational study was conducted at Dicle University, Diyarbakır, Türkiye, between 2025 and 2026. The study evaluated the effects of intravenous (IV) and oral iron replacement therapies on inflammatory markers and serum uric acid levels in premenopausal women with iron deficiency. A total of 225 participants were included: 72 patients receiving oral iron therapy, 77 patients receiving IV iron therapy, and 76 controls without iron deficiency.

Only premenopausal women aged 18 years or older were eligible. Iron deficiency was diagnosed based on hemoglobin, serum ferritin, and transferrin saturation levels. Patients with active infection, acute or chronic inflammatory or autoimmune disease, malignancy, chronic kidney disease, chronic liver disease, or another chronic systemic disorder that could affect inflammatory markers, uric acid levels, or iron metabolism were excluded. Pregnant and postmenopausal women were also excluded. Participants in the control group had no evidence of iron deficiency, anemia, active inflammation, malignancy, or chronic systemic disease.

In accordance with the institutional standard operating procedure (SOP), all patients assigned to IV iron therapy received oral paracetamol 1000 mg, intravenous methylprednisolone 20 mg, and intravenous pheniramine hydrogen maleate 45.5 mg as premedication approximately 30 min before the iron infusion.

### 2.2. Ethical Considerations

The study was conducted in accordance with the Declaration of Helsinki and approved by the Ethics Committee of Dicle University (Approval No. 282; date of approval: 24 June 2025).

### 2.3. Treatment and Follow-Up

Patients with iron deficiency received either oral or IV iron replacement according to the clinical assessment and treatment decision of the attending physician. Patients in the oral iron group were prescribed ferrous glycine sulfate complex (Ferro Sanol Duodenal), providing 100 mg of elemental iron per capsule, at a dose of one capsule twice daily (200 mg elemental iron/day), to be taken on an empty stomach. Patients in the intravenous iron group received ferric carboxymaltose, with the total replacement dose individualized according to baseline hemoglobin concentration and body weight using the recommended dosing scheme for this formulation. Doses of 500 mg were diluted in 100 mL of 0.9% sodium chloride and infused over at least 6 min, whereas doses of 1000 mg were diluted in 250 mL of 0.9% sodium chloride and infused over at least 15 min. Additional doses were administered at two-week intervals when required to complete the calculated iron replacement course. Laboratory evaluations were performed immediately before treatment and repeated on Day 30. The control group underwent baseline laboratory assessment only and did not receive iron replacement therapy. Treatment-related adverse events were prospectively assessed throughout the 30-day follow-up period and graded according to CTCAE version 5.0.

### 2.4. Laboratory Assessments

The laboratory parameters evaluated at baseline included hemoglobin, mean corpuscular volume (MCV), red cell distribution width (RDW), serum ferritin, transferrin saturation, C-reactive protein (CRP), erythrocyte sedimentation rate (ESR), serum uric acid, creatinine, and phosphorus. The same parameters were reassessed on Day 30 in the oral and IV iron groups.

The absolute change in each laboratory parameter was calculated using the following formula: Δ = Day 30 value − baseline value.

### 2.5. Sample-Size Calculation

In the absence of directly applicable prior estimates of the between-group difference in CRP change, the sample-size calculation assumed a standardized effect size of 0.50 as a planning assumption. This value was not based on an established minimum clinically important difference for CRP in this population. For a two-sided Mann–Whitney U test, with a type I error rate of 0.05, 80% statistical power, and an allocation ratio of 1:1, at least 134 evaluable patients were required. Allowing for an approximately 10% rate of missing data or loss to follow-up, the target sample size was set at 149 patients. The final analysis included 72 patients receiving oral iron and 77 patients receiving intravenous iron, fulfilling the calculated sample-size requirement.

### 2.6. Study Outcomes

The primary outcome was the change in CRP from baseline to Day 30. Changes in ESR and serum uric acid were secondary outcomes. Changes in hemoglobin, ferritin, and transferrin saturation were evaluated as additional measures of hematologic and biochemical response.

### 2.7. Statistical Analysis

IBM SPSS Statistics version 26 and Stata SE 19.5 for Windows (StataCorp, College Station, TX, USA) were used for statistical analyses. Continuous variables were expressed as median and minimum–maximum because the data did not demonstrate a normal distribution. Baseline characteristics of the control, oral iron, and IV iron groups were compared using the independent-samples Kruskal–Wallis test. When the overall comparison was statistically significant, post hoc pairwise comparisons were performed, and the resulting *p* values were adjusted using the Bonferroni correction. Day 30 laboratory values and changes from baseline to Day 30 were compared between the oral and IV iron groups using the Mann–Whitney U test. The primary comparison of CRP changes between treatment groups was performed using the Mann–Whitney U test. No multiplicity adjustment was applied to this single primary comparison. Analyses of secondary outcomes were considered exploratory, and their *p*-values were not adjusted for multiple testing. Exploratory associations between baseline CRP and hemoglobin, ferritin, and transferrin saturation were assessed using Spearman’s rank correlation analysis in the 149 patients with iron deficiency, excluding healthy controls. Additional exploratory multivariable linear regression analyses were performed to address baseline differences between the treatment groups. Separate models were fitted for Day 30 CRP, ESR, and serum uric acid, with treatment group as the exposure of interest. Each model included age, baseline hemoglobin, and the baseline value of the corresponding outcome; the serum uric acid model additionally included baseline creatinine. Heteroskedasticity-robust standard errors were used consistently across all three models. Analyses included patients receiving oral or intravenous iron and excluded healthy controls. Each model used patients with complete data for the included variables. Results are reported as adjusted mean differences between the intravenous and oral iron groups with 95% confidence intervals and two-sided *p*-values. All statistical tests were two-sided, and a *p* value of <0.05 was considered statistically significant.

## 3. Results

### 3.1. Baseline Characteristics

A total of 225 premenopausal women were included in the study: 76 participants in the control group, 72 patients in the oral iron group, and 77 patients in the intravenous (IV) iron group. Median age did not differ significantly among the three groups (33.5, 37.0, and 36.0 years, respectively; *p* = 0.054). Regarding baseline hematologic parameters and iron indices, both iron-deficient groups had significantly lower hemoglobin, mean corpuscular volume (MCV), ferritin, and transferrin saturation levels and higher red cell distribution width (RDW) than the control group (all Bonferroni-adjusted *p* < 0.001). Baseline hemoglobin was also significantly lower in the IV iron group than in the oral iron group (8.60 vs. 10.70 g/dL; adjusted *p* < 0.001), indicating more severe anemia in patients treated with IV iron. However, baseline MCV, RDW, ferritin, and transferrin saturation did not differ significantly between the two treatment groups.

**Figure 1:**
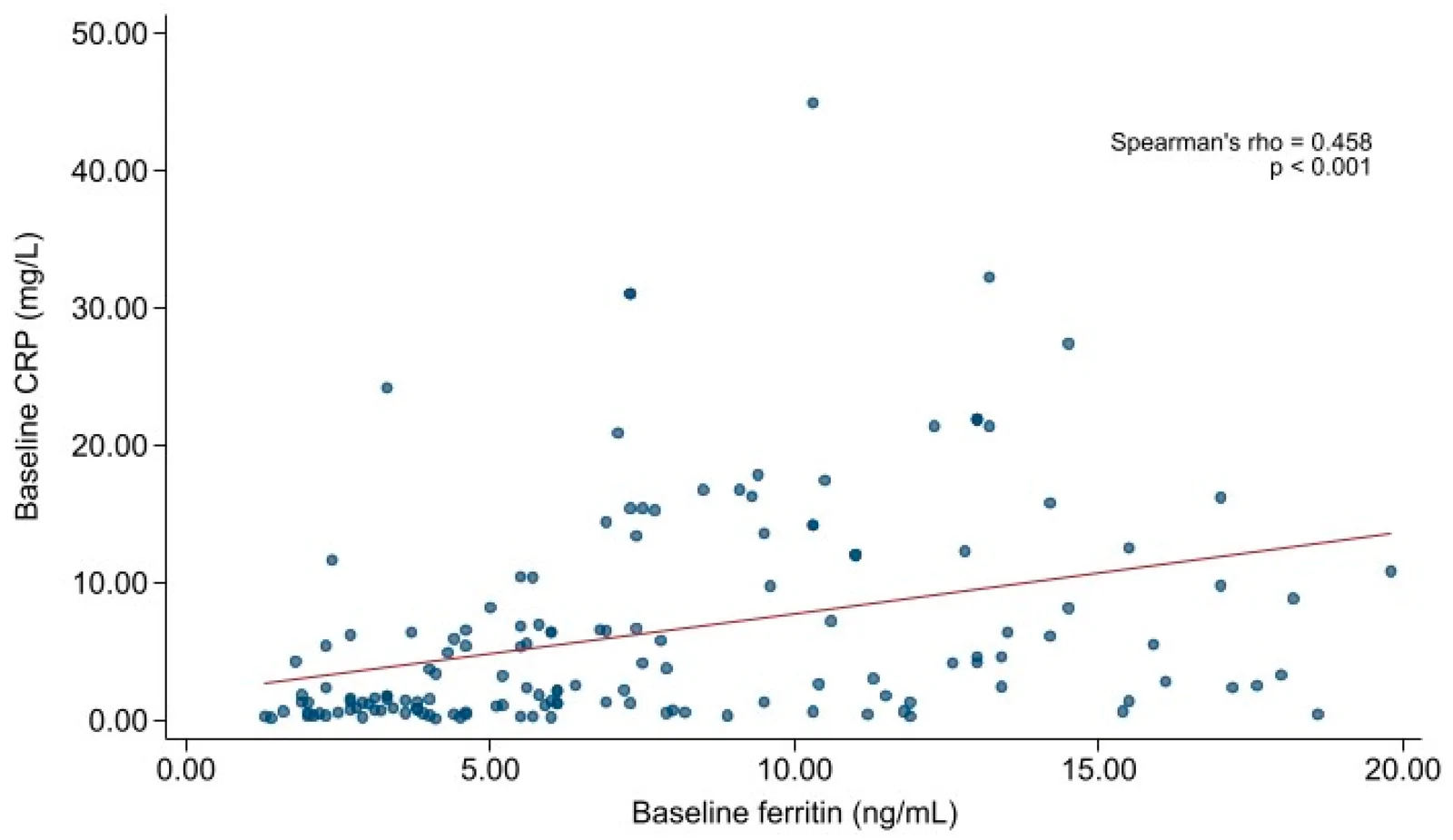
Association between baseline ferritin and C-reactive protein (CRP) in 149 premenopausal women with iron deficiency. Each point represents one patient. The solid line shows the fitted linear regression trend for visualization. The correlation coefficient and *p*-value were calculated using Spearman’s rank correlation analysis (ρ = 0.458; *p* < 0.001).

Baseline C-reactive protein (CRP) levels were significantly higher in the oral and IV iron groups than in the control group (2.96 and 2.35 vs. 0.85 mg/L, respectively; both adjusted *p* < 0.001). Similarly, erythrocyte sedimentation rate (ESR) values were higher in the oral and IV iron groups than in the control group (12.0 and 14.5 vs. 3.66 mm/h, respectively; both adjusted *p* < 0.001). Neither CRP nor ESR differed significantly between the oral and IV iron groups (both adjusted *p* = 1.000). Baseline creatinine was significantly lower in the IV iron group than in the oral iron group (0.60 vs. 0.65 mg/dL; adjusted *p* = 0.023) and the control group (0.60 vs. 0.67 mg/dL; adjusted *p* = 0.002), whereas the oral iron and control groups did not differ significantly (adjusted *p* = 1.000). Baseline uric acid and phosphorus levels did not differ significantly among the three groups (*p* = 0.365 and *p* = 0.080, respectively). Among the 149 patients with iron deficiency, baseline CRP was positively correlated with ferritin (Spearman’s ρ = 0.458; *p* < 0.001; Figure 1) and transferrin saturation (ρ = 0.236; *p* = 0.004). The correlation between CRP and hemoglobin was weakly negative and did not reach statistical significance (ρ = −0.156; *p* = 0.057) (Table 1 and Table 2). At Day 30, the relationship between CRP and ferritin was also assessed in the overall treatment cohort and separately within each treatment group. No statistically significant correlations were observed in the overall cohort (Spearman’s ρ = 0.019; *p* = 0.814; *n* = 149), the oral iron group (ρ = 0.089; *p* = 0.455; *n* = 72), or the intravenous iron group (ρ = 0.170; *p* = 0.140; *n* = 77).

### 3.2. Day 30 Laboratory Findings

At Day 30, median hemoglobin levels increased to 12.50 g/dL in the oral iron group and 12.00 g/dL in the IV iron group, with no statistically significant difference between the groups (*p* = 0.072). MCV values were also comparable (*p* = 0.824). RDW was slightly higher in the IV iron group than in the oral iron group (15.80% vs. 14.75%; *p* = 0.024). RDW data at Day 30 were available for 70 patients in the oral iron group and 72 patients in the IV iron group. Iron stores were significantly higher following IV iron treatment. At Day 30, median ferritin was 123.10 ng/mL in the IV iron group compared with 15.80 ng/mL in the oral iron group (*p* < 0.001). Similarly, median transferrin saturation was higher in the IV iron group than in the oral iron group (35.68% vs. 15.61%; *p* < 0.001). Day 30 CRP levels did not differ significantly between the IV and oral iron groups (2.23 vs. 3.52 mg/L; *p* = 0.089). However, ESR was significantly lower in the IV iron group than in the oral iron group (10.0 vs. 12.0 mm/h; *p* = 0.040). Serum uric acid at Day 30 was significantly higher in the IV iron group than in the oral iron group (4.80 vs. 4.10 mg/dL; *p* = 0.015). No significant differences were observed in creatinine or phosphorus levels between the groups (*p* = 0.562 and *p* = 0.500, respectively) (Table 3).

### 3.3. Changes from Baseline to Day 30

The increase in hemoglobin was significantly greater in the IV iron group than in the oral iron group (median Δ: 3.00 vs. 1.75 g/dL; *p* < 0.001). The increases in ferritin and transferrin saturation were also substantially greater with IV iron. Median ferritin increased by 115.80 ng/mL in the IV iron group and by 5.60 ng/mL in the oral iron group (*p* < 0.001), while the corresponding increases in transferrin saturation were 23.18 and 5.87 percentage points, respectively (*p* < 0.001). The change in CRP differed significantly between the treatment groups. The median change in CRP was −0.22 mg/L in the IV iron group and +0.07 mg/L in the oral iron group (*p* = 0.041). Although this difference was statistically significant, its absolute magnitude was small. Changes in ESR did not differ significantly between the oral and intravenous iron groups (median Δ: 0.00 mm/h in both groups; *p* = 0.199). The median change in serum uric acid was 0.00 mg/dL in the oral iron group and 0.20 mg/dL in the intravenous iron group, with no statistically significant between-group difference (*p* = 0.228) (Table 4).

### 3.4. Adjusted Analysis

After adjustment for age, baseline hemoglobin, and baseline CRP, the estimated Day 30 CRP level was lower in the intravenous iron group, but the difference was not statistically significant (adjusted difference, IV minus oral: −1.585 mg/L; 95% CI, −3.261 to 0.091; *p* = 0.064; *n* = 149). In the corresponding ESR model, the intravenous group had a significantly lower Day 30 ESR after adjustment for age, baseline hemoglobin, and baseline ESR (adjusted difference: −2.118 mm/h; 95% CI, −3.817 to −0.418; *p* = 0.015; *n* = 91). For serum uric acid, adjustment for age, baseline hemoglobin, baseline uric acid, and baseline creatinine yielded a positive but non-significant between-group difference (adjusted difference: 0.389 mg/dL; 95% CI, −0.009 to 0.787; *p* = 0.055; *n* = 149). All confidence intervals and *p*-values were calculated using heteroskedasticity-robust standard errors.

### 3.5. Safety

No adverse events, including infusion-related reactions, were observed among patients receiving IV iron. In the oral iron group, no grade ≥2 gastrointestinal adverse events were reported during the 30-day follow-up period. Serum phosphorus was assessed at baseline and Day 30. At Day 30, median phosphorus levels were 3.54 mg/dL (range, 1.17–6.76) in the oral iron group and 3.35 mg/dL (range, 1.19–6.63) in the intravenous iron group, with no statistically significant between-group difference (*p* = 0.500).

## 4. Discussion

The principal finding of this study was that the greater hematologic and biochemical response observed with intravenous iron was not accompanied by a consistent difference across inflammatory markers. The modest between-group difference in CRP change contrasted with the absence of a significant difference in ESR change, suggesting that these markers may reflect different aspects of the response to iron replacement. This pattern warrants cautious interpretation rather than being considered evidence of a general anti-inflammatory advantage of intravenous over oral iron.

Randomized trials in women with heavy uterine bleeding or postpartum iron-deficiency anemia have compared intravenous ferric carboxymaltose with oral ferrous sulfate. These studies demonstrated greater hematologic responses and more effective iron-store repletion with intravenous ferric carboxymaltose over six weeks [14,15]. Nevertheless, comparative evidence regarding the early effects of oral and IV iron on systemic inflammation and uric acid metabolism remains limited. Unlike previous studies, we simultaneously evaluated changes in CRP, ESR, and serum uric acid during the first month of oral and IV iron treatment in otherwise healthy premenopausal women with iron deficiency.

The higher baseline CRP and ESR levels in patients with iron deficiency than in controls should not be interpreted as evidence that iron deficiency itself caused systemic inflammation. Inflammation can restrict iron availability through hepcidin-mediated mechanisms, while anemia and erythrocyte characteristics can influence ESR independently of inflammatory activity [4,5,6]. Although patients with recognized infectious, inflammatory, and chronic systemic diseases were excluded, unrecognized inflammatory conditions and factors related to the underlying cause of iron deficiency cannot be completely ruled out. The baseline comparisons therefore demonstrate an association but do not establish its direction or causality.

The exploratory correlation analysis provided additional context for interpreting baseline CRP levels. Within the iron-deficient cohort, CRP correlated positively with ferritin and, more weakly, with transferrin saturation, whereas its association with hemoglobin did not reach statistical significance. These findings do not support a simple pattern in which higher CRP consistently accompanies more severe iron depletion or anemia. However, these unadjusted associations cannot determine the direction of the relationship or establish that CRP elevation is independent of iron status. The positive association with transferrin saturation also warrants cautious interpretation and further investigation rather than being attributed to a specific mechanism.

One possible explanation for the modest CRP change is that correction of iron deficiency may influence inflammatory signaling in addition to restoring erythropoiesis. Experimental work in iron-deficient mice has linked low hepcidin levels to an enhanced inflammatory response to lipopolysaccharide, suggesting a potential interaction between iron availability and innate immune regulation [7]. Clinical observations also provide some support for this possibility: Bankir and Yanardağ Açık [8] reported a reduction in CRP after iron replacement in women with iron-deficiency anemia. More efficient iron repletion could therefore be one possible contributor to the CRP pattern observed in the intravenous group. However, hepcidin and inflammatory cytokines were not measured in our study, and this mechanistic explanation remains speculative.

The absolute CRP changes were small, with median changes of −0.22 mg/L in the intravenous group and 0.07 mg/L in the oral group. Their clinical relevance therefore remains uncertain despite the statistically significant between-group comparison in the unadjusted analysis. Changes in this small magnitude may reflect biological fluctuation or analytical variation rather than a clinically meaningful alteration in inflammatory activity. Our study cannot distinguish these possibilities, and statistical significance in the unadjusted comparison should not be interpreted as evidence of an anti-inflammatory benefit. In the additional analysis adjusted for baseline CRP, baseline hemoglobin, and age, the Day 30 CRP difference was not statistically significant (adjusted difference, IV minus oral: −1.585 mg/L; 95% CI, −3.261 to 0.091; *p* = 0.064). Although these analyses address different comparisons, the adjusted findings further limit the interpretation of the small unadjusted difference as evidence of clinical benefit. Treatment allocation was not randomized, and the intravenous group had more severe anemia at baseline; adjustment for measured covariates cannot eliminate residual confounding by treatment indication or disease severity. Moreover, all patients in the intravenous group received methylprednisolone, paracetamol, and pheniramine before infusion, whereas patients in the oral group did not receive this premedication regimen. Premedication was therefore inseparable from treatment-group assignment, and adjustment for baseline characteristics could not distinguish the effect of intravenous iron from that of the accompanying medications. The contribution of premedication to Day 30 CRP, ESR, and serum uric acid measurements cannot be determined from our data. The comparisons should consequently be understood as associations with the treatment regimens as administered, rather than isolated effects of the route of iron administration. At Day 30, CRP and ferritin were not significantly correlated in the overall treatment cohort or within either treatment group. These exploratory findings provide additional context but neither establish nor exclude a direct inflammatory or anti-inflammatory effect of iron therapy. The observed CRP changes cannot be attributed specifically to iron administration because treatment allocation was nonrandomized and premedication was administered exclusively to the intravenous group. The sample-size calculation was based on an assumed standardized effect size rather than an established clinically meaningful CRP difference. Consequently, the study was not designed to provide a definitive assessment of a prespecified clinically important treatment effect. The small observed difference should therefore be interpreted cautiously, regardless of its statistical significance in the unadjusted analysis.

The differing CRP and ESR findings may reflect the distinct biological determinants of these markers. CRP is a hepatic acute-phase protein that responds relatively rapidly to inflammatory stimuli, whereas ESR is an indirect measure influenced by plasma proteins and erythrocyte number, size, and morphology. Anemia can therefore affect ESR independently of systemic inflammation, and changes in the erythrocyte population during iron replacement may complicate its interpretation [6]. Discordance between CRP and ESR has also been documented in clinical populations [16]. Accordingly, a small change in CRP need not be accompanied by a parallel change in ESR. Nevertheless, their different response kinetics alone cannot explain our Day 30 findings, and measurements at only baseline and Day 30 do not allow the temporal relationship between hematologic recovery and inflammatory marker changes to be established.

Although ESR was statistically lower in the intravenous group at Day 30, the difference between the group medians was only 2 mm/h (10 versus 12 mm/h). This comparison describes ESR levels at a single follow-up time point and does not establish that intravenous iron produced a greater reduction from baseline. The absence of a significant between-group difference in ESR change further limits that interpretation. Differences in baseline ESR distributions and hematologic recovery may contribute to the apparent discrepancy between the follow-up and change-score comparisons. No clinically meaningful threshold for ESR change was prespecified, and the observed difference was not linked to a clinical outcome. Its clinical significance therefore remains uncertain and should not be interpreted as evidence of superior inflammatory control with intravenous iron.

The adjusted analyses further qualified the interpretation of the unadjusted findings. Although the unadjusted comparison of CRP changes was statistically significant, the adjusted Day 30 CRP comparison did not meet the conventional threshold for statistical significance. The adjusted serum uric acid difference was also not statistically significant. In contrast, Day 30 ESR remained lower in the intravenous group after adjustment for baseline ESR, hemoglobin, and age. This finding indicates an association after accounting for the measured covariates, but the approximately 2 mm/h difference has uncertain clinical significance. Residual confounding and corticosteroid premedication restricted to the intravenous group preclude attributing these associations solely to the route of iron administration.

A substantial body of evidence suggests that serum uric acid is associated with systemic inflammation and may serve as an indirect inflammation-related metabolic biomarker. Higher uric acid concentrations have been associated with conventional inflammatory markers and activation of the NF-κB and NLRP3 inflammasome pathways [9,10]. In a previous study by Dal et al. [17], patients with immune thrombocytopenia had significantly higher serum uric acid levels than healthy controls, and uric acid was positively correlated with CRP, supporting its potential relationship with systemic inflammatory activity. However, evidence regarding uric acid specifically in iron deficiency and following iron replacement remains limited. Remacha et al. [12] reported a significant increase in serum uric acid after four months of oral iron therapy in young women with iron-deficiency anemia. In our study, serum uric acid was higher in the intravenous group at Day 30 in the unadjusted comparison. However, the additional analysis adjusted for baseline uric acid, hemoglobin, creatinine, and age did not demonstrate a statistically significant between-group difference (IV minus oral: 0.389 mg/dL; 95% CI, −0.009 to 0.787; *p* = 0.055). Thus, the unadjusted Day 30 difference alone does not establish a route-specific effect on serum uric acid, and the adjusted findings should not be interpreted as evidence of equivalence between the treatment regimens.

The 30-day follow-up was intended to assess early treatment responses and may not capture the full hematologic response or restoration of iron stores with oral therapy. Consequently, the greater early repletion observed with intravenous iron should not be interpreted as evidence of superior long-term efficacy. Quantitative treatment-adherence data were unavailable, limiting assessment of actual oral iron exposure and its contribution to the observed differences. Safety assessment was also limited to this follow-up period. Phosphorus measurements were obtained only at baseline and Day 30; therefore, transient hypophosphatemia occurring between these assessments could not be excluded.

In conclusion, intravenous iron was associated with greater increases in hemoglobin, ferritin, and transferrin saturation than oral iron over 30 days. However, the modest between-group difference in CRP change and the absence of a significant difference in ESR change do not establish a clinically meaningful anti-inflammatory advantage of intravenous iron. The lower ESR level in the intravenous group at Day 30 should be interpreted cautiously, given the small absolute difference and the lack of a corresponding difference in changes from baseline. The observational design, baseline differences between treatment groups, and limited follow-up preclude causal conclusions. Larger randomized studies with serial measurements of inflammatory and metabolic markers are needed to clarify these relationships.

## Figures and Tables

**Table 1:** Baseline demographic and laboratory characteristics of the study groups.

Variable	Control (*n* = 76)	Oral Iron (*n* = 72)	IV Iron (*n* = 77)	Overall *p*
Age, years	33.50 (19.00–45.00)	37.00 (18.00–45.00)	36.00 (18.00–45.00)	0.054
Hemoglobin, g/dL	13.60 (13.00–15.50)	10.70 (8.00–11.90)	8.60 (5.70–11.90)	*<0.001*
MCV, fL	87.50 (80.20–94.20)	74.00 (64.70–79.00)	73.00 (65.70–79.10)	*<0.001*
RDW, %	13.00 (11.20–14.80)	14.65 (12.30–20.80)	15.30 (11.33–24.40)	*<0.001*
Ferritin, ng/mL	31.95 (21.20–188.80)	6.25 (1.80–19.80)	6.10 (1.30–17.00)	*<0.001*
Transferrin saturation, %	29.84 (21.00–44.26)	9.65 (2.80–19.03)	9.40 (2.03–19.85)	*<0.001*
CRP, mg/L	0.85 (0.01–9.76)	2.96 (0.11–24.20)	2.35 (0.17–44.94)	*<0.001*
Sedimentation, mm/hour	3.66 (2.88–22.00)	12.00 (4.00–44.00)	14.50 (3.00–30.00)	*<0.001*
Uric acid, mg/dL	4.30 (2.30–6.40)	4.00 (2.10–9.90)	4.30 (1.60–9.30)	0.365
Creatinine, mg/dL	0.67 (0.40–1.15)	0.65 (0.29–1.16)	0.60 (0.27–1.19)	*0.002*
Phosphorus, mg/dL	3.67 (1.71–5.37)	3.42 (1.87–5.15)	3.84 (1.50–7.52)	0.080

Values are presented as median (minimum–maximum). Overall comparisons among the three groups were performed using the Kruskal–Wallis test. Bonferroni-adjusted pairwise comparisons are presented in Table 2. *p* values shown in italic font indicate statistically significant results (*p* < 0.05). MCV: mean corpuscular volume; RDW: red cell distribution width; CRP: C-reactive protein.

**Table 2:** Post hoc pairwise comparisons of baseline parameters among the three study groups.

Variable	Comparison	Adj. *p*
Hemoglobin, g/dL	IV Iron vs. Oral Iron	*<0.001*
	IV Iron vs. Control	*<0.001*
	Oral Iron vs. Control	*<0.001*
MCV, fL	IV Iron vs. Oral Iron	0.462
	IV Iron vs. Control	*<0.001*
	Oral Iron vs. Control	*<0.001*
RDW, %	Control vs. Oral Iron	*<0.001*
	Control vs. IV Iron	*<0.001*
	Oral Iron vs. IV Iron	1.000
Ferritin, ng/mL	IV Iron vs. Oral Iron	1.000
	IV Iron vs. Control	*<0.001*
	Oral Iron vs. Control	*<0.001*
Transferrin saturation, %	IV Iron vs. Oral Iron	1.000
	IV Iron vs. Control	*<0.001*
	Oral Iron vs. Control	*<0.001*
CRP, mg/L	Control vs. Oral Iron	*<0.001*
	Control vs. IV Iron	*<0.001*
	Oral Iron vs. IV Iron	1.000
Sedimentation, mm/hour	Control vs. Oral Iron	*<0.001*
	Control vs. IV Iron	*<0.001*
	Oral Iron vs. IV Iron	1.000
Creatinine, mg/dL	IV Iron vs. Oral Iron	*0.023*
	IV Iron vs. Control	*0.002*
	Oral Iron vs. Control	1.000

Pairwise comparisons were performed following a statistically significant Independent-Samples Kruskal–Wallis test; *p* values were adjusted for multiple comparisons using the Bonferroni correction. Variables for which the overall Kruskal–Wallis test was not significant (age, uric acid, phosphorus) are not shown, as post hoc comparisons are not applicable. *p* values shown in italic font indicate statistically significant results (*p* < 0.05). MCV: mean corpuscular volume; RDW: red cell distribution width; CRP: C-reactive protein.

**Table 3:** Laboratory values at Day 30 of treatment: Oral iron vs. IV iron.

Variable (Day 30)	Oral Iron (*n* = 72)	IV Iron (*n* = 77)	*p* *
Hemoglobin, g/dL	12.50 (9.00–14.90)	12.00 (9.00–15.60)	0.072
MCV, fL	85.70 (63.40–98.70)	86.80 (62.10–113.30)	0.824
RDW, %	14.75 (12.00–23.00)	15.80 (11.70–26.30)	*0.024*
Ferritin, ng/mL	15.80 (2.70–390.30)	123.10 (5.90–1343.90)	*<0.001*
Transferrin saturation, %	15.61 (3.01–120.94)	35.68 (3.85–235.97)	*<0.001*
CRP, mg/L	3.52 (0.18–24.80)	2.23 (0.06–36.46)	0.089
Sedimentation, mm/hour	12.00 (4.00–32.00)	10.00 (3.00–21.00)	*0.040*
Uric acid, mg/dL	4.10 (1.70–9.20)	4.80 (1.60–8.10)	*0.015*
Creatinine, mg/dL	0.63 (0.09–1.70)	0.65 (0.23–1.14)	0.562
Phosphorus, mg/dL	3.54 (1.17–6.76)	3.35 (1.19–6.63)	0.500

Values are presented as median (minimum−maximum). Comparisons between the two groups were performed using the Mann–Whitney U test. * *p* values shown in italic font indicate statistically significant results (*p* < 0.05). MCV: mean corpuscular volume; RDW: red cell distribution width; CRP: C-reactive protein.

**Table 4:** Change (Δ) in laboratory parameters from baseline to Day 30: Oral iron vs. IV iron.

Variable (Δ, Baseline → Day 30)	Oral Iron (*n* = 72)	IV Iron (*n* = 77)	*p* *
Δ Hemoglobin, g/dL	1.75 (−1.20–6.10)	3.00 (−0.57–6.70)	*<0.001*
Δ Ferritin, ng/mL	5.60 (−3.30–382.30)	115.80 (−2.50–1337.00)	*<0.001*
Δ CRP, mg/L	0.07 (−16.16–22.46)	−0.22 (−31.04–14.56)	*0.041*
Δ Sedimentation, mm/hour	0.00 (−40.00–4.00)	0.00 (−20.00–4.00)	0.199
Δ Transferrin saturation, % points	5.87 (−10.03–117.48)	23.18 (−13.73–224.12)	*<0.001*
Δ Uric acid, mg/dL	0.00 (−2.90–2.60)	0.20 (−5.40–4.14)	0.228

Values are presented as median (minimum–maximum) of the change (Δ = Day 30 − baseline) for each parameter. Comparisons between the two groups were performed using the Mann–Whitney U test. * *p* values shown in italic font indicate statistically significant results (*p* < 0.05). CRP: C-reactive protein; TS: transferrin saturation.

## Data Availability

The data that support the findings of this study are available from the corresponding author upon reasonable request.
